# One stage correction via the Hi-PoAD technique for the management of severe, stiff, adolescent idiopathic scoliosis curves > 90°

**DOI:** 10.1007/s43390-023-00663-4

**Published:** 2023-02-22

**Authors:** Cesare Faldini, Giovanni Viroli, Francesca Barile, Marco Manzetti, Marco Ialuna, Matteo Traversari, Fabio Vita, Alberto Ruffilli

**Affiliations:** Department of Biomedical and Neuromotor Science-DIBINEM, 1st Orthopaedic and Traumatologic Clinic, IRCCS Istituto Ortopedico Rizzoli, University of Bologna, Via Giulio Cesare Pupilli 1, 40136 Bologna, Italy

**Keywords:** Idiopathic adolescent scoliosis, Pedicle screw accuracy, Pedicle screw malpositioning, Pre-operative CT, Scoliosis

## Abstract

**Study design:**

Retrospective cohort study.

**Purpose:**

to assess the efficacy and safety of Hi-PoAD technique in patients with a major thoracic curve > 90°, < 25% of flexibility and deformity spread over more than five vertebral levels.

**Methods:**

retrospective review of AIS patients with a major thoracic curve (Lenke 1–2–3) > 90°, with < 25% of flexibility and deformity spread over more than five vertebral levels. All were treated via the Hi-PoAD technique. Radiographic and clinical score data were collected pre-operatively, operatively, at 1 year, 2 years and at last follow-up (2 years minimum).

**Results:**

19 patients were enrolled. A 65.0% correction rate of the main curve was achieved, from 101.9° to 35.7° (*p* < 0.001). The AVR reduced from 3.3 to 1.3. The C7PL/CSVL reduced from 1.5 to 0.9 cm (*p* = 0.013). Trunk Height increased from 31.1 to 37.0 cm (*p* < 0.001). At the final follow-up no significant changes, except from an improvement in C7PL/CSVL (from 0.9 cm to 0.6 cm; *p* = 0.017). SRS-22 increased in all patients, from 2.1 to 3.9 at 1 year of follow-up (*p* < 0.001). 3 patients had a transient drop of MEP and SEP during maneuver and were managed with temporary rods and a second surgery after 5 days. 2 of these 3 cases (66.7%) had a Total-Deformity Angular Ratio (T-DAR) > 25; conversely, among patients who had a one-stage procedure, only 1 (6.2%) had a T-DAR > 25 (*p* = 0.008).

**Conclusions:**

The Hi-PoAD technique proved to be a valid alternative for the treatment of severe, rigid AIS involving more than 5 vertebral bodies.

**Study design:**

Retrospective comparative cohort study.

**Level of evidence:**

III.

## Introduction

The surgical treatment of severe rigid adolescent idiopathic scoliosis (AIS) is still highly debated [1–3]. One-stage Posterior-only spinal fusion (PSF) has historically been the mainstay of AIS treatment, however, achieving an adequate triplanar correction of this kind of curves may be challenging. Therefore, combined antero-posterior spinal fusions [1, 2], have been proposed to gain a more powerful correction, but the high rate of pulmonary complications remains cause of concern [3]. Staged corrections have therefore gained wide spread, using pre-operative halo traction, either standalone [4], or after anterior [5–8] or posterior [9] releases. These techniques, although effective, are really demanding for the patients and lead to significant health-related costs, considering the prolonged period of hospitalization and the multiple surgical procedures required. To offer a better tolerated alternative to these patients, a temporary internal distraction with magnetically controlled growing rods (MCGR) [10, 11] has been proposed; however, increased concerns about its cost-effectiveness may arise due to the addition of high hardware costs. One-staged correction using aggressive tricolumnar osteotomies (3COs) such as vertebral column resection (VCR [12–14] represents an effective alternative, but at a cost of high technical complexity, alongside an high complications rate.

All the above-mentioned techniques proved to be precious and effective, but each particular procedure should be applied according to the pathological anatomy of the deformity. The Authors’ hypothesis is that severe and stiff AIS with deformity spread over more than five vertebral levels should be effectively treated by a technique named *Hi-PoAD* (High Density Pedicle screws, Ponte Osteotomies, Asymmetric Rods Contouring, Direct Vertebral Rotation), already described for the management of rigid Adult AIS [15]. The rationale is to perform a safe and effective one-stage posterior procedure, exerting strong corrective forces by a combination of different maneuvers, via an aggressive release and high-density constructs. The aim of this article is to present the results of *Hi-PoAD* technique for the treatment of these curves, at minimum 2 years of follow-up, focusing on its efficacy and safety.

## Materials and methods

### Study sample

A retrospective review of AIS patients with a major thoracic curve (Lenke 1, 2, 3 patterns) above 90°, with a side bending (SB) reduction < 25% and with major curve spread over more than 5 vertebral bodies, who underwent surgery with *Hi-PoAD* technique, was undertaken. Non-idiopathic scoliosis, infantile or juvenile idiopathic scoliosis diagnosis were considered exclusion criteria. Follow-up evaluations were performed post-operatively, at 1 and 2 years and up to final follow-up. All patients signed an informed consent on the use of their clinical documentation for scientific purposes. The local Ethics Committee approved this retrospective study.

### Data collection

Operative time, blood loss [both as an absolute volume (EBL) and as a percentage of total blood volume (%EBV, estimated as 75 ml multiplied by the body weight)], length of stay, intra- and post-operative complications were recorded. Coronal Cobb angle of each curve, coronal flexibility index (difference in Cobb angle of the main curve between the pre-operative full-length standing and lateral supine side-bending X-rays, expressed as a percentage), T5–T12 thoracic kyphosis (TK) and L1–S1 lumbar lordosis (LL) were measured. Apical vertebral rotation (AVR) was measured according to Nash-Moe. Total Deformity Angular Ratio (T-DAR; the sum of Coronal and Sagittal DAR, which are calculated as the Cobb angle divided by the number of vertebrae involved in the curve) was evaluated. C7 plumb line (C7PL)/central sacral vertical line (CSVL) and sagittal vertical axis (SVA) were used to assess coronal and sagittal balance. Trunk Height (TH) was measured as the distance between the midpoint of the upper endplate of T1 and the midpoint of the lower endplate of the LIV, to evaluate the spinal height. SRS-22 questionnaire was administered pre-operatively and at 1 year of follow-up.

### Surgical technique

**Fig. 1:**
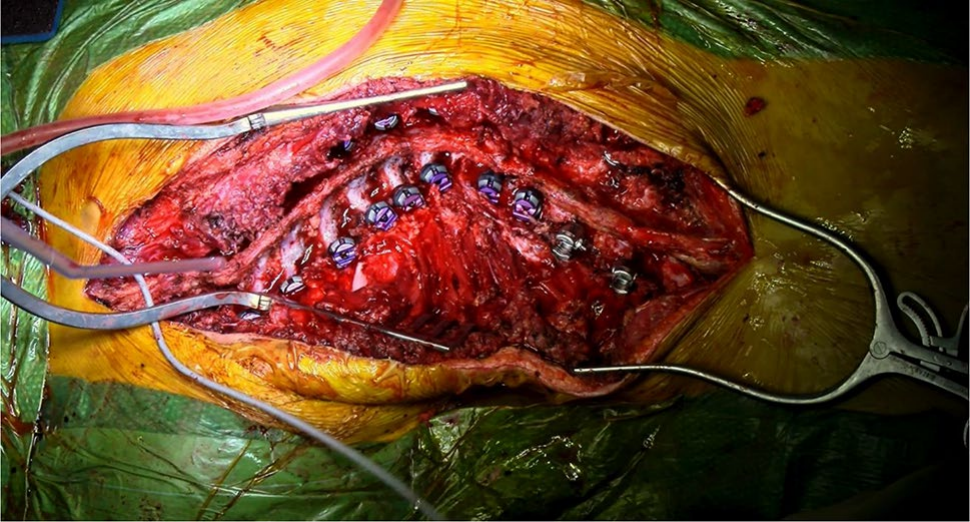
Exemplificative surgical image of a high-density pedicle screw construct: T3–L2 fusion with screws placed at all pedicles, except for left T10 pedicle

**Fig. 2:**
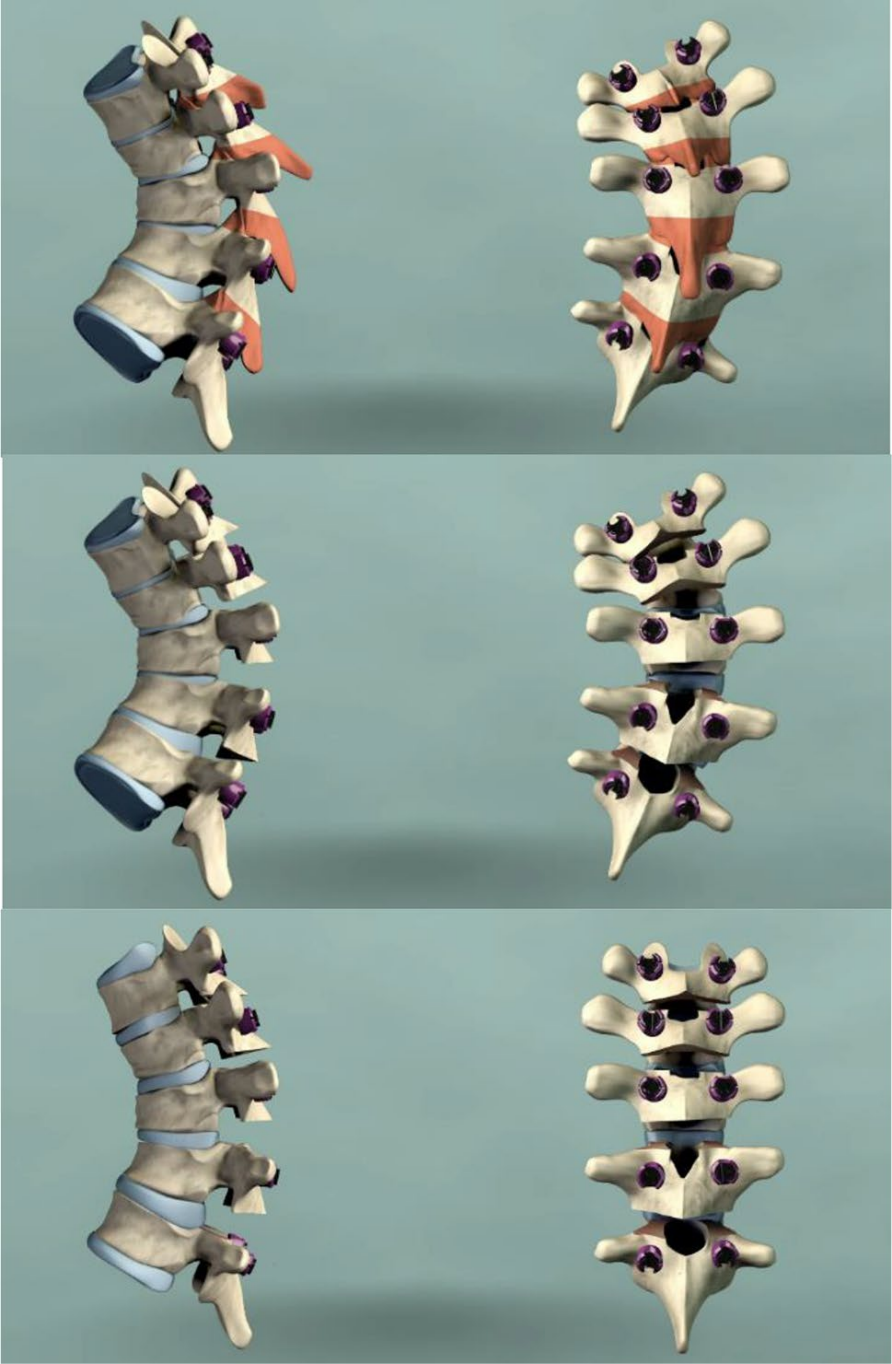
Ponte osteotomies: exemplificative drawing

**Fig. 3:**
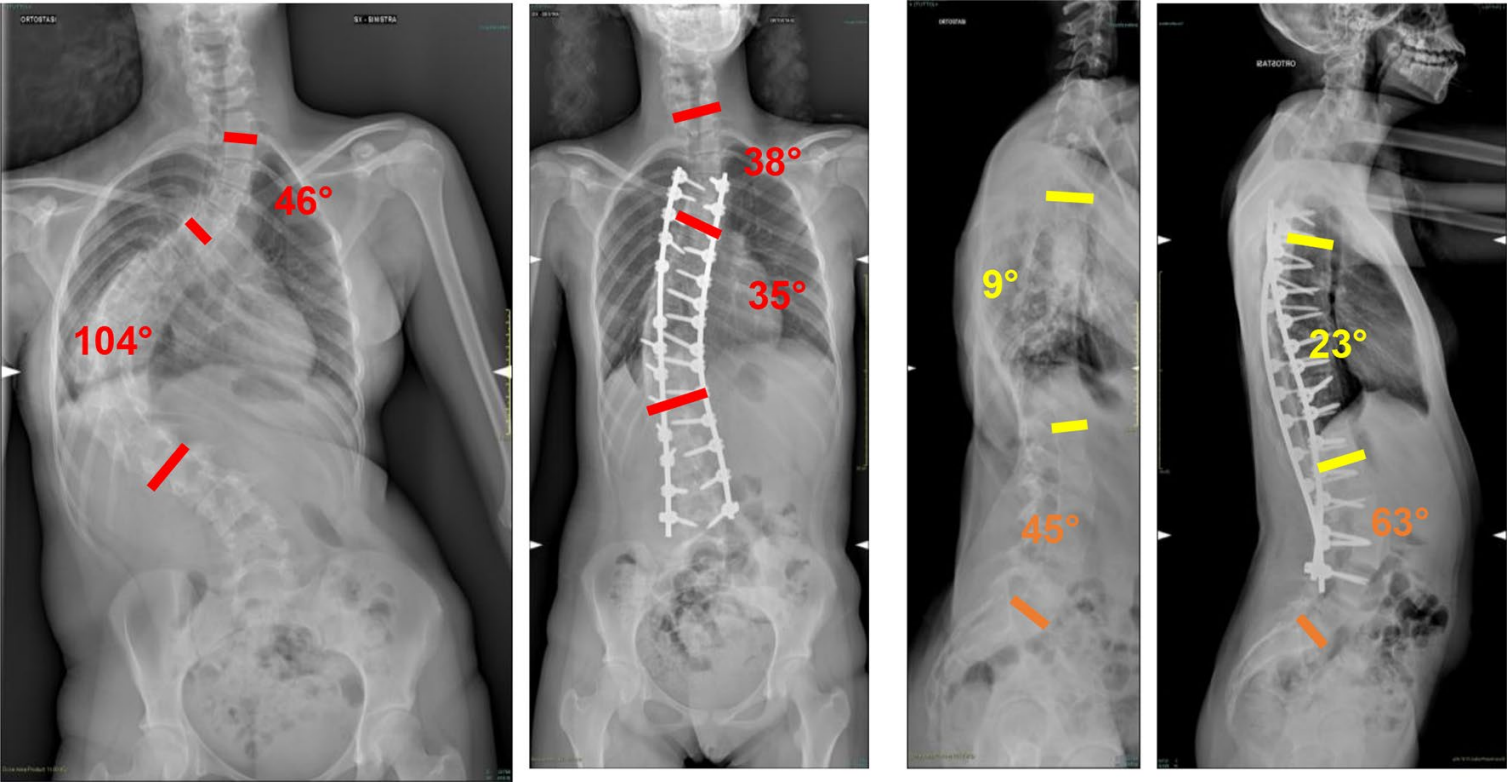
Case 1, a 17 years old female with a Lenke 2 curve, treated by T4–L4 fusion; pre and post-operative X-rays

**Fig. 4:**
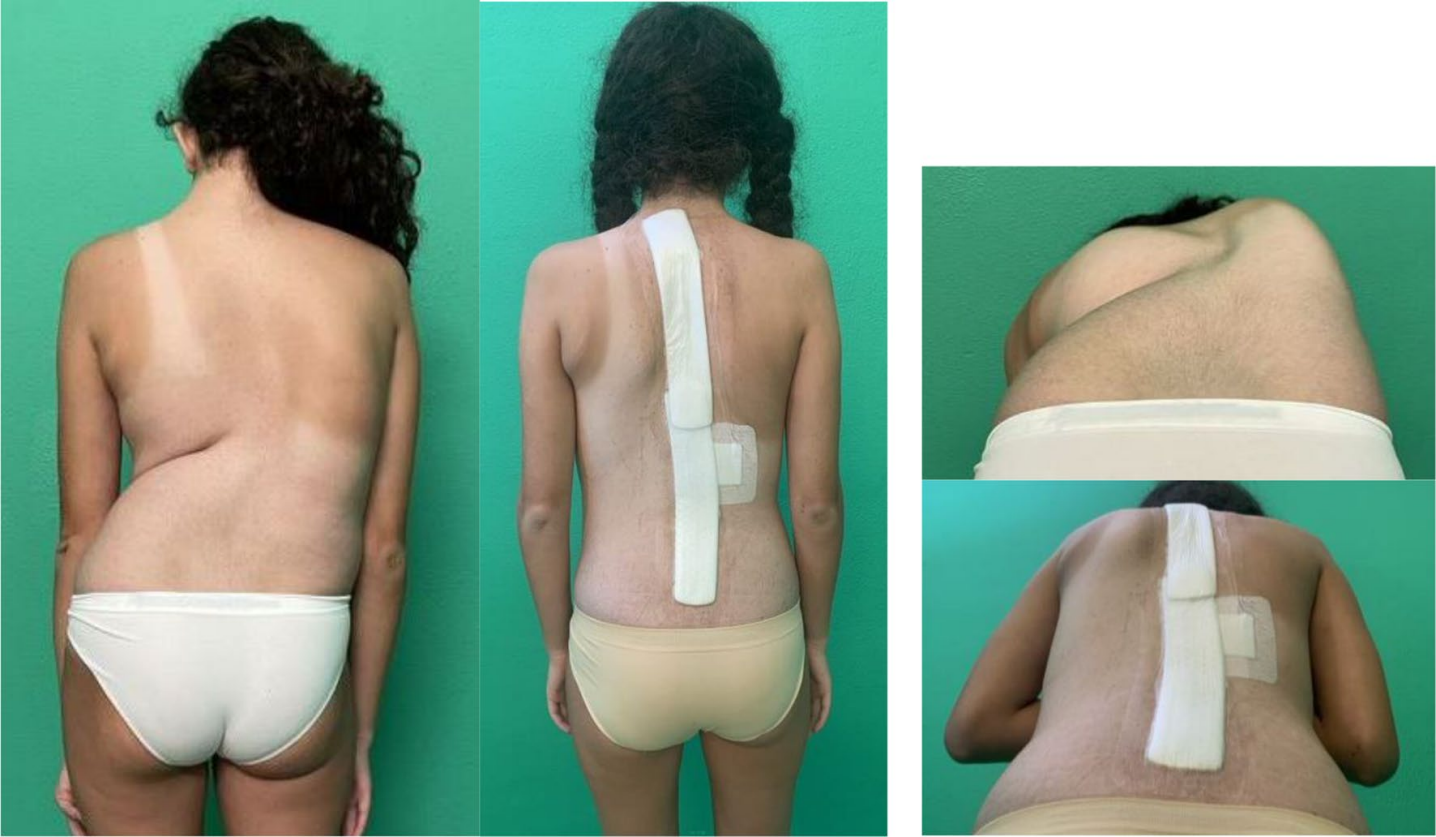
Case 1, a 17 years old female with a Lenke 2 curve, treated by T4–L4 fusion; pre and post-operative clinical appearance

**Fig. 5:**
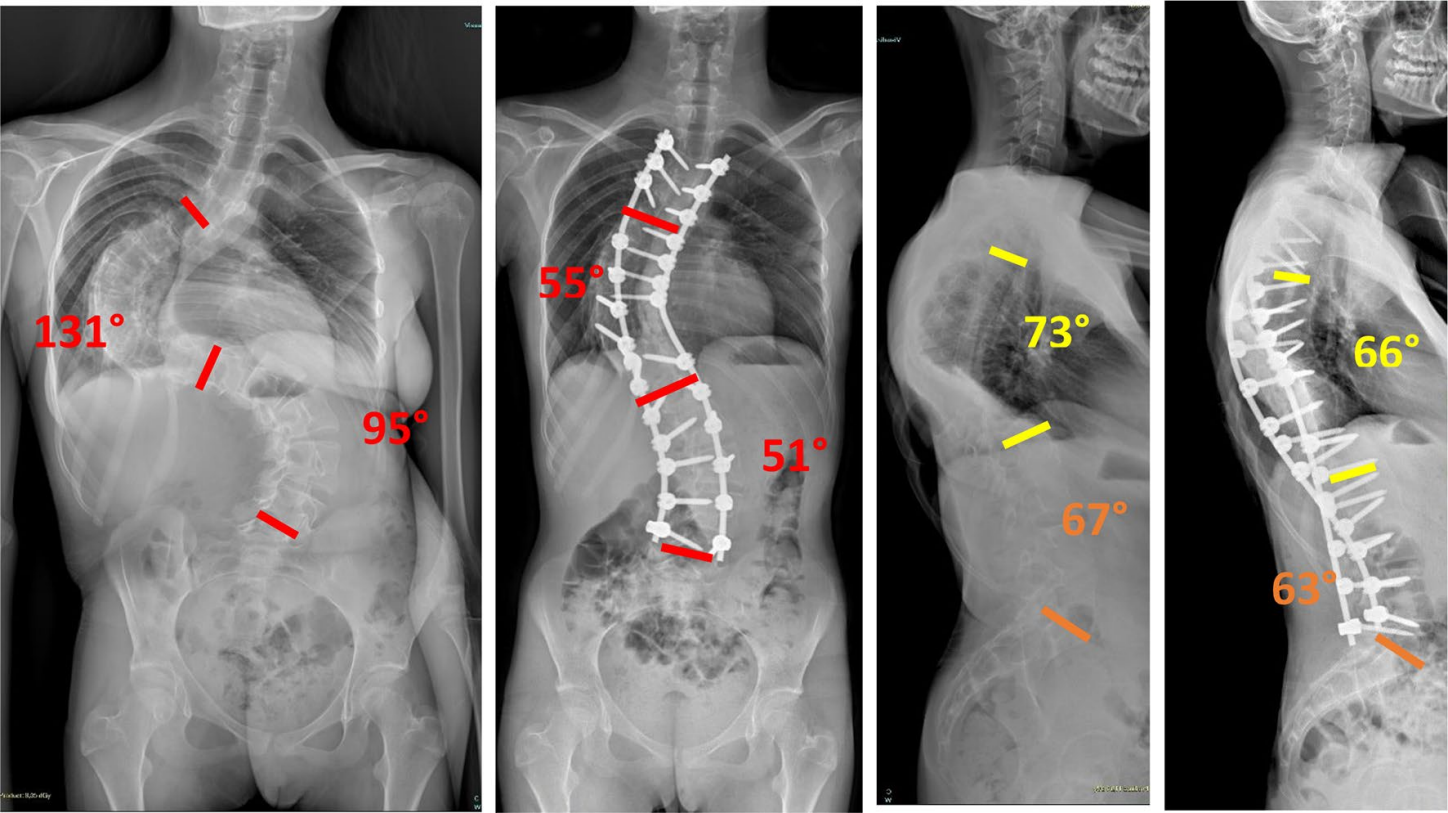
Case 3, a 13 years old female with a Lenke 3 curve, treated with a T3–L5 fusion; pre- and post-operative X-rays

**Fig. 6:**
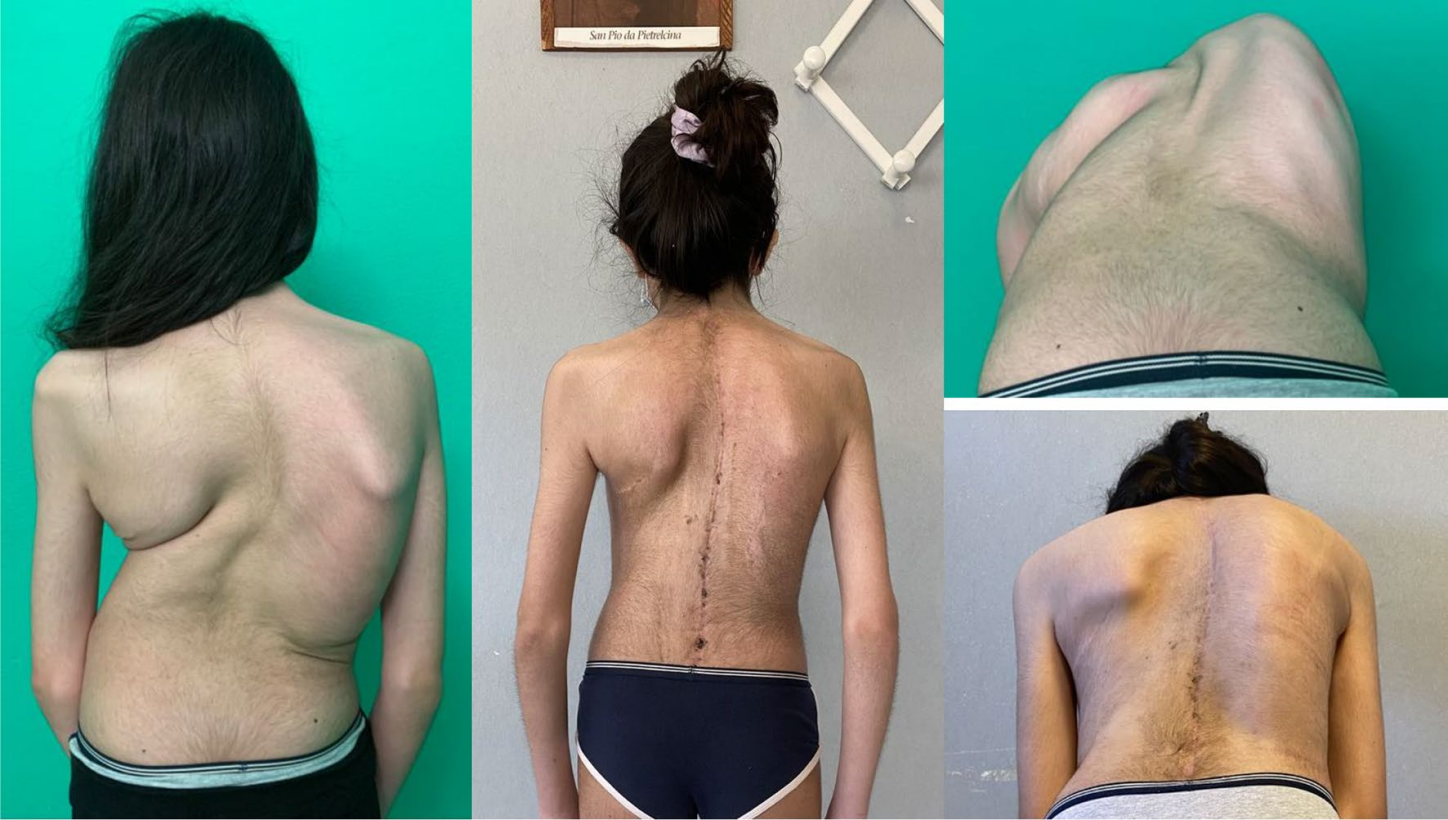
Case 3, a 13 years old female with a Lenke 3 curve, treated with a T3–L5 fusion; pre- and post-operative clinical appearance

**Fig. 7:**
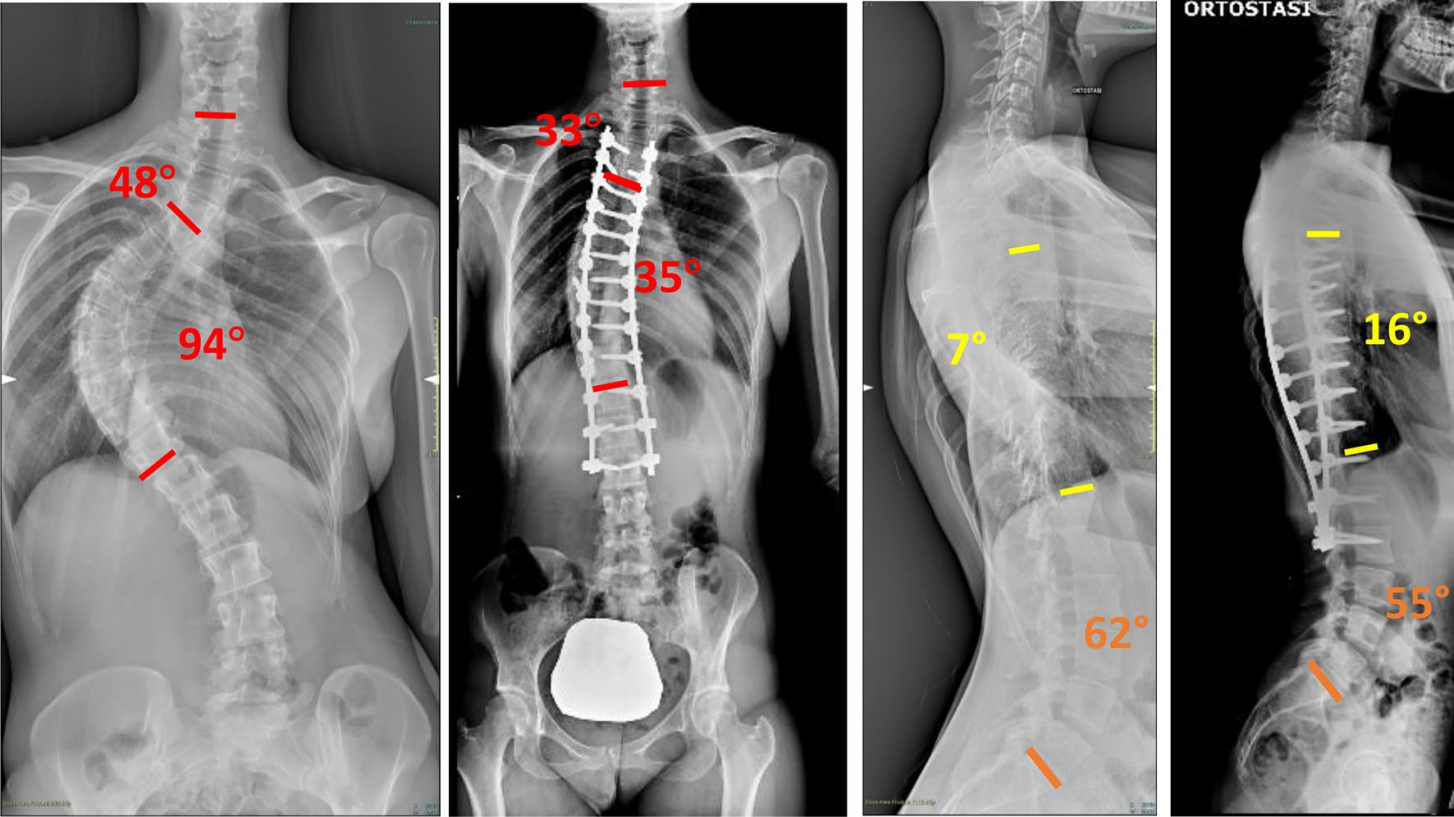
Case 4, a 18 years old female with a Lenke 2 curve, treated with a T3–L3 fusion; pre- and post-operative X-rays

**Fig. 8:**
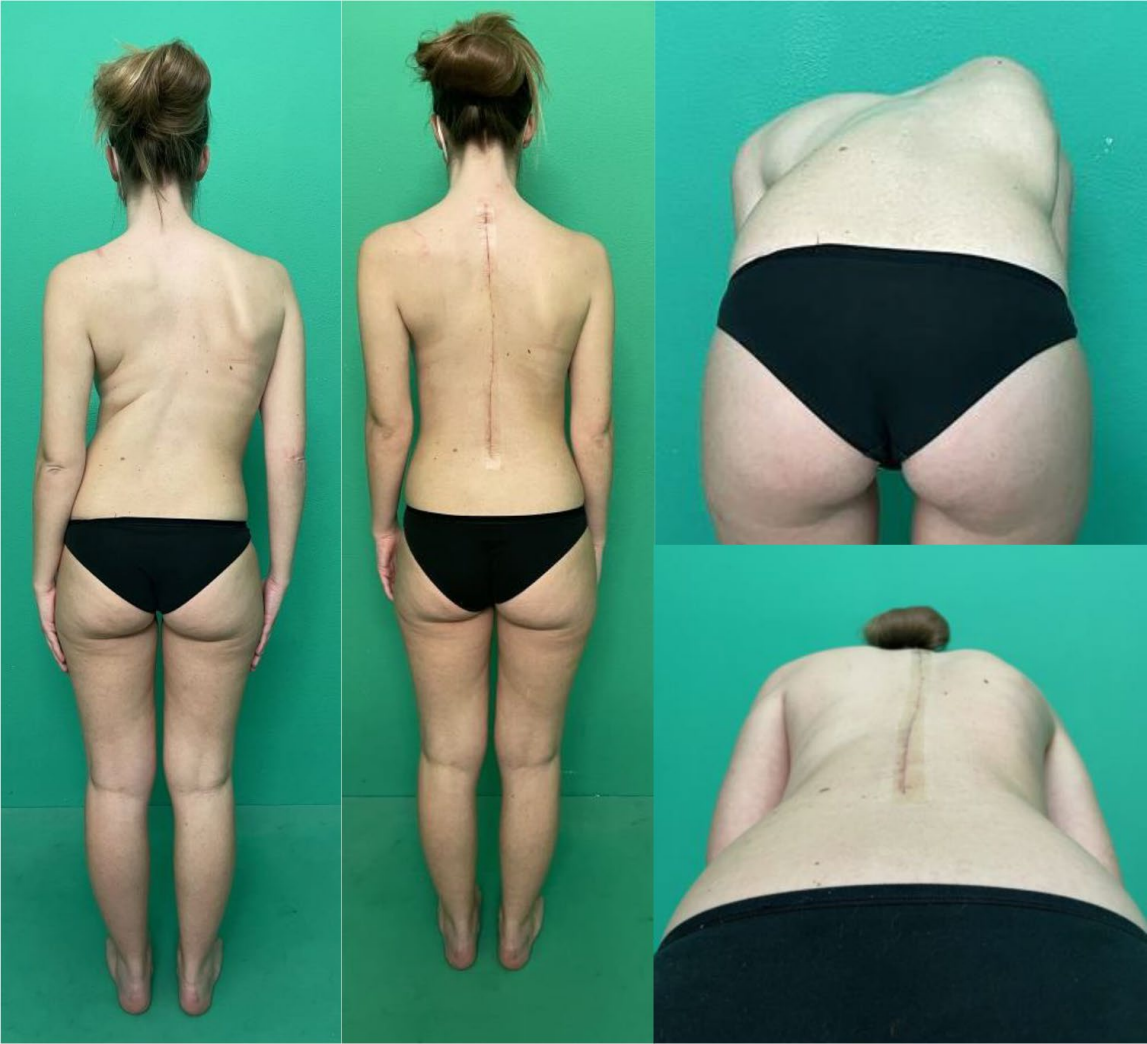
Case 4, a 18 years old female with a Lenke 2 curve, treated with a T3–L3 fusion; pre- and post-operative clinical appearance

All surgeries were performed by the first author, according to the Hi-PoAD technique [15]. The fusion area was defined according to the criteria by Lenke et al. [16]. All pedicle screws were placed with the free-hand, power-assisted technique [17]. To optimize the transmission of forces from the tulip to the screw, and therefore to the vertebral body, all-uniplanar-screws constructs were preferred in all cases (Fig. 1). Differently from the original technique [15] the first Ponte osteotomy (Fig. 2) was performed at the apex of the main curve and at the four periapical levels (two above and two below the apex) in all cases, to maximize the corrective potential. In Lenke 3 cases, 5 Ponte or Smith-Petersen osteotomies were performed at thoracolumbar-lumbar curve apex if curve magnitude exceeded 90°. Two 5.5-mm cobalt-chrome rods were asymmetrically molded, to achieve a synergic action on sagittal profile restoration, with a lifting effect at the physiological apex of thoracic kyphosis and a pushing effect in the lumbar spine to restore lumbar lordosis; at the thoracic scoliosis apex, the concavity rod was higher (over-bended) than the convexity rod, to obtain an indirect derotational effect. The rods were passed through each anchor point and simultaneously rotated with the curves consensually oriented on the sagittal plane. Rotation tubes were applied at apical screws and at the LIV to exert derotational forces and counter-torque forces respectively, both in convexity and concavity. Keeping derotational forces applied, the translation maneuver was progressively performed. Before the definitive engage of the rods in the tulips, selective compression and distraction were performed at apical and periapical levels to close gaps at the Ponte osteotomy sites. Final engagement of the rods was then performed. In situ coronal or sagittal bending was then performed if needed. An extensive decortication of the posterior elements of the instrumented vertebrae was performed to obtain a solid fusion bed, followed by apposition of autologous and homologous bone. Subfascial drain was placed, and the wound was closed in multiple layers, with running intradermal suture for the skin. Exemplificative cases are presented (Figs. 3, 4, 5, 6, 7, 8).

Early mobilization started on post-operative day 1. For the first 4 weeks after surgery, a thoracic lumbar sacral orthosis (TLSO) to restrict spinal movements and facilitate initial bone graft fusion was prescribed.

### Patients characteristics

19 (16 females and 3 males) were included, with a follow-up of 29.2 ± 3.1 (range 28–37). The average age was 13.7 ± 1.6 (range 12–18). Patient’s characteristics are summarized in Table 1.

**Table 1:** Patients' characteristics

Patients *n*	19 (16 females; 3 males)
Average age (years)	13.7 ± 1.6 (range 12–18)
Curve type	I: 8; II: 4; III: 6; IV 1
T-DAR	18.6 ± 6.1 (range 14–33.7)
Average fused levels	13.1 ± 1.4 (range 10–15)
Average pedicle screws implanted	24.5 ± 2.2 (range 20–29)
Screw density	94.2% ± 5.5% (range 83.3–100%)
Average surgical time (minutes)	263.1 ± 39.1 (range 226–364)
Average blood loss (ml)	892.7 ± 137.3 (range 546–1032)
Average blood loss (%EBV)	21.8 ± 3.7% (range 14.3–26.7%)
Average follow-up (months)	29.2 ± 3.1 (range 28–37)
Average length of stay (days)	7.9 ± 4.1 (range 5–19)

### Statistical analysis

Parametric test was used to compare samples in case of continuous variables, normal distribution and appropriate numerousness. The Shapiro–Wilk test was used to verify normal distribution. As parametric test, two-tailed student t test was used to compare the average of the variables for homoscedastic paired groups. Pearson's chi-squared test was applied to compare categorical data. Continuity correction was applied in case of discrete distribution. *p* values < 0.05 were considered to be significant. Furthermore, Intraclass Correlation Coefficient (ICC) and Cohen’s Kappa were used to assess intra- and inter-observer agreement in the measurement of pre-operative and last follow-up values of TK and AVR, respectively. Jamovi statistical analysis software (The jamovi project (2021), jamovi Version 1.6) was used to perform statistical analysis.

## Results

Average surgical time was 263.1 ± 39.1 min, and average blood loss was 892.7 ± 137.3 (21.8 ± 3.7% EBV) (Table 1). Patients were discharged after an average length of stay of 7.9 ± 4.1 (range 5–19).

An effective triplanar correction was achieved (Table 2): the average post-operative Cobb angle decreased by 65.0% (from 101.9° ± 14.2° to 35.7° ± 15.9°, *p* < 0.001). On the sagittal plane, both post-operative TK and LL remained stable, passing from 24.7° ± 21.8° to 26.3° ± 15.3° (+ 6.5%; *p* = 0.454) and from 51.9° ± 15.3° to 51.0° ± 11.6° (− 1.7%; *p* = 0.710) respectively. The average AVR significantly reduced from 3.3 ± 0.7 to 1.3 ± 0.5 (− 60.6%; *p* < 0.001). The average C7PL/CSVL reduced with statistical significance from 1.5 cm ± 0.8 to 0.9 cm ± 0.6 (− 40.0%; *p* = 0.013). TH significantly increased from 31.1 cm ± 3.5 to 37.0 cm ± 3.4 (+ 19.0%; *p* < 0.001). At the final follow-up no significant differences were noted in the radiological results compared to post-operative values, except from a significative improvement in C7PL/CSVL (from 0.9 cm ± 0.6 to 0.6 cm ± 0.3; − 33.3% *p* = 0.017). Intra- and inter-observer agreement showed good agreement for TK both in pre-operative and last follow-up measurements, while AVR did record substantial agreement in the pre-operative measurements and only moderate agreement in the last FU measurements (Table 3).

**Table 2:** Average values of the examined variables pre-operatively, post-operatively, at 1-year, 2-years and last follow-up

	Pre-op	Post-op	% variation pre-op vs post-op	*p* value pre-op vs post-op	1-year FU	2-years FU	Last FU	% variation pre-op vs last FU	*p* value pre-op vs last FU	% variation post-op vs last FU	*p* value post-op vs last FU
Main coronal curve	101.9° ± 14.2°	35.7° ± 15.9°	− 65.0	< 0.001*	35.9° ± 15.5°	36.1° ± 15.6°	36.4° ± 15.3	− 64.3	< 0.001*	+ 1.9	0.114
Coronal flexibility	− 17% ± 5.7%	–	–	–	–		–	–	–	–	–
TK	24.7° ± 21.8	26.3° ± 15.3	+ 6.5	0.454	26.5° ± 14.5	26.8° ± 15.1	26.7° ± 14.4	+ 8.1	0.362	+ 1.5	0.143
LL	51.9° ± 15.3	51.0° ± 11.6	− 1.7	0.710	51.3° ± 11.6	51.6° ± 11.7	51.7° ± 11.6	− 0.4	0.931	+ 1.4	0.184
AVR	3.3 ± 0.7	1.3 ± 0.5	− 60.6	< 0.001*	1.4 ± 0.5	1.4 ± 0.5	1.4 ± 0.5	− 57.6	< 0.001*	+ 7.7	0.330
C7PL/CSVL	1.5 cm ± 0.8	0.9 cm ± 0.6	− 40.0	0.013*	0.8 cm ± 0.4	0.7 cm ± 0.3	0.6 cm ± 0.3	− 60.0	< 0.001*	− 33.3	0.017*
TH	31.1 cm ± 3.5	37.0 cm ± 3.4	+ 19.0	< 0.001*	36.9 cm ± 3.4	36.8 cm ± 3.5	36.9 cm ± 3.3	+ 18.7	< 0.001*	+ 3.4	0.091
SRS-22	2.1 ± 0.5	–	–	–	3.9 ± 0.4	–		+ 85.7	< 0.001*^,^^	–	–

*FU* follow-up, *TK* thoracic kyphosis, *LL* lumbar lordosis, *C7PL/CSVL* C7 plumb-line (C7PL)/central sacral vertical line (CSVL), *AVR* apical vertebral rotation according to Nash-Moe, *TH* trunk height, *SRS-22* Scoliosis Research Society 22-item questionnaire

*Statistically significant

^SRS-22 was administered pre-op and at the 1-year FU, so for SRS-22 “last FU” is considered the 1 year-FU

**Table 3:** Intra and interobserver agreement for AVR and TK in the pre-operative and last follow-up measurements

TK pre-op	Intra-rater ICC 1	0.978 (95% CI 0.943–0.987)	Excellent
	Intra-rater ICC 2	0.982 (95% CI 0.956–0.992)	Excellent
	Inter-rater ICC	0.970 (95% CI 0.929–0.983)	Excellent
TK last FU	Intra-rater ICC 1	0.979 (95% CI 0.899–0.992)	Excellent
	Intra-rater ICC 2	0.971 (95% CI 0.892–0.983)	Excellent
	Inter-rater ICC	0.935 (95% CI 0.766–0.966)	Excellent
AVR pre-op	Intra-rater Cohen’s kappa 1	0.796 (95% CI 0.534–1.000)	Substantial
	Intra-rater Cohen’s kappa 2	0.735 (95% CI 0.456–1.000)	Substantial
	Inter-rater Cohen’s kappa	0.635 (95% CI 0.320–0.949)	Substantial
AVR last FU	Intra-rater Cohen’s kappa 1	0.692 (95% CI 0.396–0.988)	Substantial
	Intra-rater Cohen’s kappa 2	0.553 (95% CI 0.176–0.930)	Moderate
	Inter-rater Cohen’s kappa	0.469 (95% CI 0.073–0.866)	Moderate

ICC interpretation: below 0.50: poor, between 0.50 and 0.75: moderate, between 0.75 and 0.90: good, above 0.90: excellent. Kappa interpretation: between 0.00 and 0.20: Slight agreement, between 0.21 and 0.40: Fair agreement, between 0.41 and 0.60: Moderate agreement, between 0.61 and 0.80: Substantial agreement, between 0.81 and 1.00: Almost perfect agreement

SRS-22 questionnaire score increased in all patients, from a pre-operative value of 2.1 ± 0.5 to a mean of 3.9 ± 0.4 at 1 year of follow-up (*p* < 0.001).

**Fig. 9:**
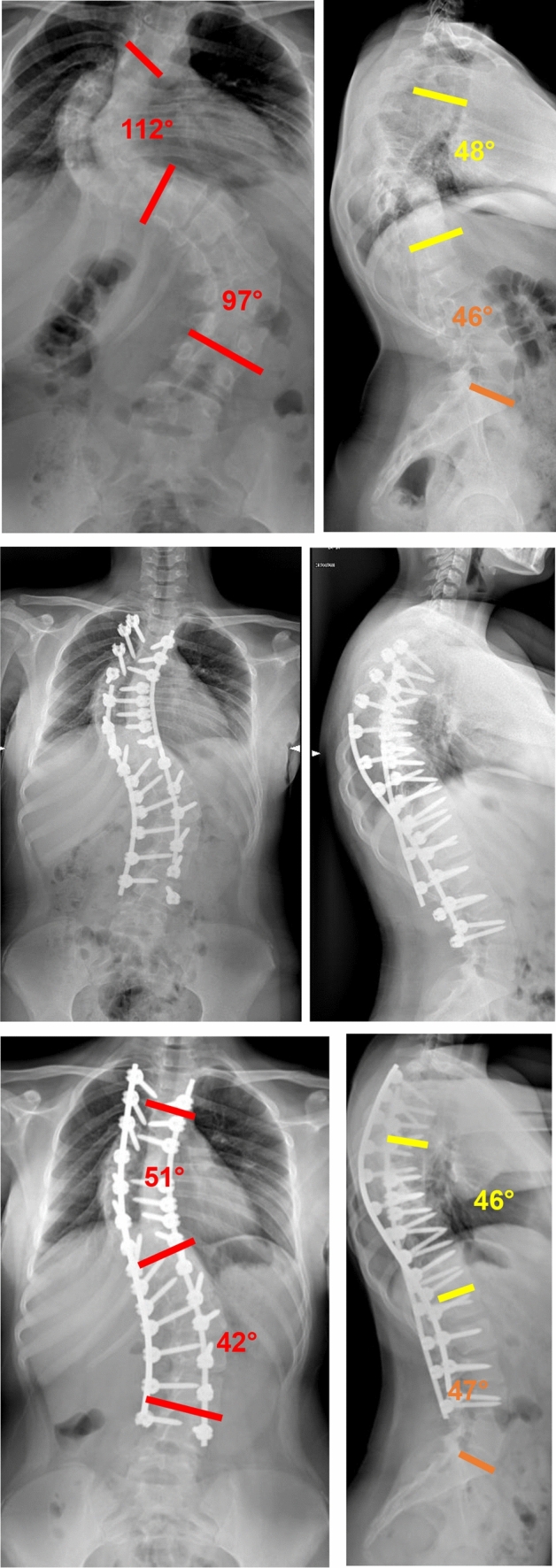
Case 2, a 15 years old female with a Lenke 3 curve, treated by T3–L4 fusion. The patient had a transient SEP and MEP drop during corrective maneuver, so the patient was temporarily stabilized with two titanium rods and managed with a second stage surgery after 5 days. Pre-operative and post-operative (first and second stage surgery) X-rays

**Fig. 10:**
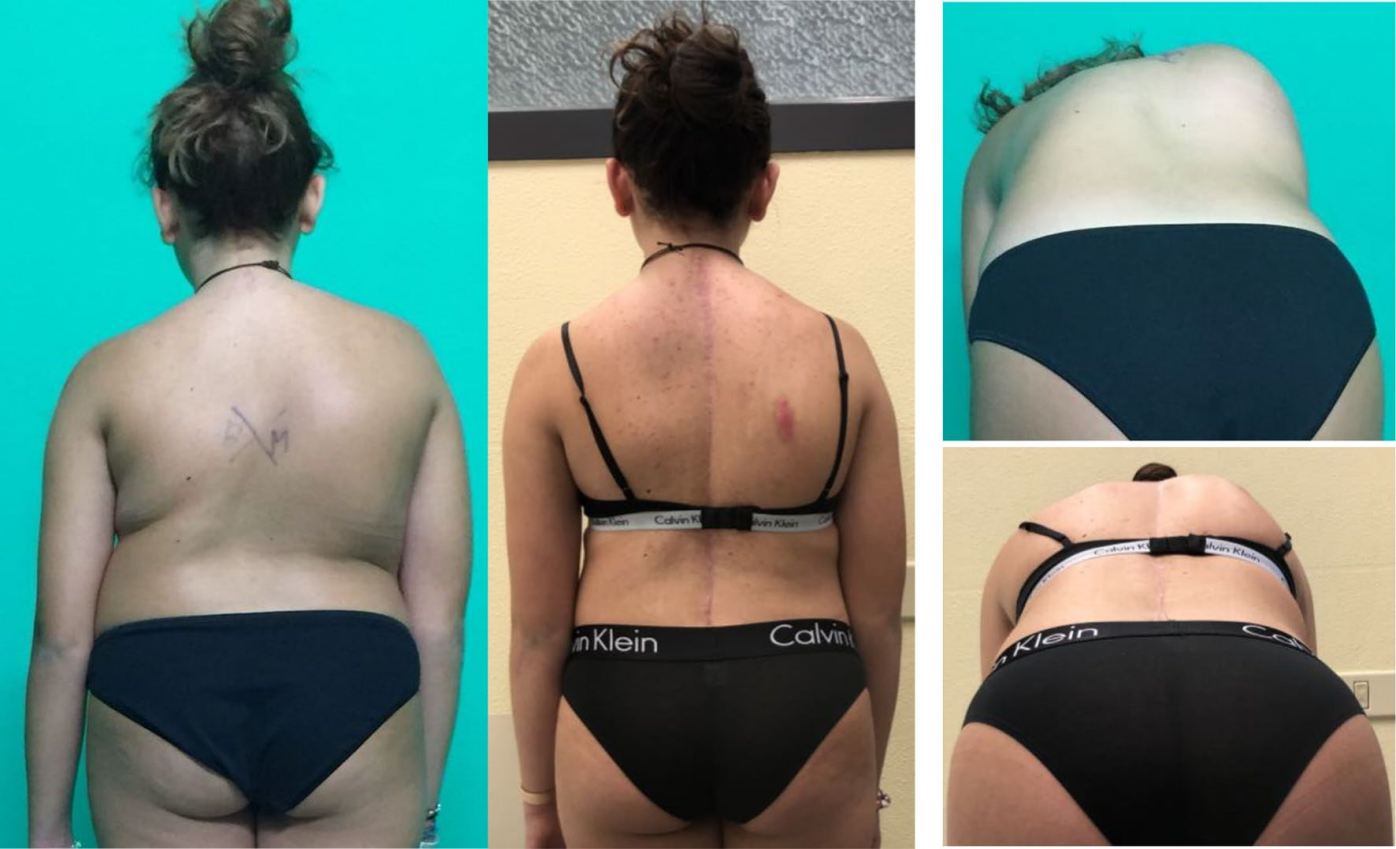
Case 2, a 15 years old female with a Lenke 3 curve, treated by T3–L4 fusion; pre and Postoperative clinical appearance

3 patients had an intraoperative SEP and MEP reduction during the corrective maneuver. In these cases, a counter-maneuver was performed and the mean arterial pressure was raised, to enhance the recovery of the evoked potentials. Once the recovery happened, a temporary rod was placed in slight distraction on the concavity of the main curve (and on the concavity of the lumbar curve in Lenke 3 cases) to stabilize the spine in a partial corrected position and surgery was interrupted. The definitive surgery was performed 5 days later, to allow the spinal cord to get used to the new corrected spinal alignment (Figs. 9, 10). 2 of these 3 cases (66.7%) had a T-DAR > 25; conversely, among patients who had a one-stage procedure, only 1 (6.2%) had a T-DAR > 25 (*p* = 0.008) (Table 4). No mechanical complications (screws pull-out, pedicle breakage, necessity to extend the fusion area) were registered intraoperatively.

**Table 4:** Relationship between T-DAR and surgical stages required for correction

T-DAR	1-stage	2-stage	TOTAL
> 25	1 (6.2%)	2 (66.7%)	3 (23.1%)
< 25	15 (93.8%)	1 (33.3%)	16 (76.9%)
TOTAL	16 (100%)	3 (100%)	19 (100%)
*p*: 0.008*			

*Stastically significant

At the last follow-up, no mechanical nor infective complications were registered; no cases of correction loss, adding on or junctional pathology were observed.

## Discussion

The described technique proved to be really efficient, allowing a powerful coronal correction rate of 65.0%, which is superior compared to the results reported by others all-posterior one-stage correction studies without 3COS [18–20] (50.9–55.9%). This difference can be explained by the combination of corrective strategies that the described technique provides. First, an extremely high screw density with screws placed at every level permits to dissipate corrective forces, thus limiting pull-out risks. Secondly, the aggressive release based upon periapical Ponte Osteotomies enforces the translation and derotational maneuverers allowing in addition segmental correction with convex compression and concave distraction. Thirdly, the simultaneous application of the asymmetrically molded CoCr rods allows to apply a translational and derotational force over both rods at the same time, improving the overall corrective capacity. Finally, for the coupling principle, DVR and translation maneuvers act simultaneously: the DVR has a translation component that acts in synchrony with the translation performed over the rods; on the other side, the asymmetric shape of the rods exerts an indirect derotational force that combines with DVR. This combination of strategies, that come at the cost of an increased technical complexity, allowed also a good triplanar correction of the deformity, respecting a physiological sagittal profile (no significant changes in TK and LL), reducing the vertebral body rotation (significant AVR reduction) and restoring a correct coronal balance (significant C7PL-CSVL reduction). In particular, when looking at TK, our results are in line with a growing body of literature that reports a better control of kyphosis when combining pedicle screws and Ponte osteotomies [21, 22]. The addition of Asymmetric Rods Contouring to high-density screws systems, may further make possible the respect of TK even when large amounts of coronal and axial plane corrections are needed.

It may be underlined that despite the complexity of the technique average surgical time and blood loss resulted in line with the majority of the case series dealing with all posterior techniques without 3COS (263.1 min vs 180.5–300 min and 892.7 ml vs 850–1752 ml respectively [18–20]).

Some authors [23, 24] reported a correction rate as high as 77% for severe scoliosis curves, adopting a staged technique based upon anterior release. In fact, anterior release allows to achieve a direct control over the anterior disk restraint to DVR. On the contrary, the presented technique, as it is based on an all-posterior approach, allows to exert powerful corrective forces which may overcome the anterior disk restraints only indirectly and/or partially. Despite that, the morbidity related to the anterior approach may outweigh the benefit of the additional correction.

The correction rate achieved via the Hi-PoAD technique was comparable to that of staged procedures based on traction (both internal and external) (65.0% vs 51.3–67.4% [4–8, 10, 11]), but without the need of prolonged challenging traction periods, multiple procedures and high health- and hardware-related costs. The rationale of staged procedures is to obtain a gradual distraction resulting in adequate correction minimizing stresses over the spinal cord. Looking in detail at the safety profile of the techniques, Hi-PoAD reported a 15.8% rate of transient intraoperative MEP/SEPs deterioration, without any sequalae at the wakening. However, it must be acknowledged that even staged procedures are not exempt from neurologic risks, with a reported rate of 0–14.2% % [4–8, 10, 11], ranging from transient MEPs reduction [10], to transient [4, 6, 7] and permanent [5] motor deficit. This seem to imply that the spinal cord risks during correction may be dependent upon other factors besides the correction techniques, such as curve intrinsic characteristics. In our cohort, a T-DAR > 25 significantly correlated with the need to perform a 2-stage procedure due to SCM changes during the first correction attempt. On one hand, this seems to support the higher risks of SCM events reported by Wang et al. [25] for patients with a T-DAR > 25. On the other hand, there are certainly other factors acting that still need to be identified, since the patient with the most severe DAR (33.7) of our series still had a one-stage procedure, while one patient with a 22.3 DAR had a two-stage procedure. So, given the unpredictability and the generally transitory nature of SCM events during a cautious and progressive corrective manouver, in addition to the relatively high success rate of the procedure (84.2%), in our experience a one-stage Hi-PoAD procedure may still be suitable for these curves. Moreover, this strategy remained cost-effective even in the 3 cases where a staged procedure was required, given the fact that relatively economic hardware was used (simple titanium temporary rod vs MCGR) and that hospital stay was relatively short (13–19 days) in these 3 cases; certainly shorter compared to internal or external traction techniques [4, 6–11].

The correction rate of the presented technique was similar to that achieved by VCR studies (65.0% vs 61–67% [12–14]). However, the Hi-PoAD technique allowed significantly lower mean EBL (892.7 ml vs 1610–7034 ml [12–14]), general complications rate (0% vs 25–59% [12–14]) and neurological complications rate (15.8%, all transient, vs 0–27% [12–14]), compared to VCR. Moreover, an often-neglected issue with these patients is that they usually have a mismatch between trunk height and leg length due to the severity of the spinal deformity. The use of VCR, which is a shortening procedure, may therefore result in failure to restore a proper trunk height/leg length ratio compared the increase of TH achieved by the presented technique. For all these reasons, in the Authors’ opinion, VCR remains a fundamental weapon, but with limited indications such as: extremely sharp deformities, often with a kyphotic component and associated myelopathy. In these situations, the powerful translation between the proximal and distal vertebral sections, and the circumferential spinal cord decompression that it offers, are still unmatched by any other technique while it may be too aggressive for severe AIS curves involving more than 5 vertebral bodies.

The results of the present study must be seen in light of some important limitations. First, the definition of severe scoliosis is still unclear, with studies using as cutoff a Cobb angle > 80° [1, 5, 12], others > 90° [2, 11, 14, 23] and others > 100° [10, 24]. The definition of rigid curve is even more confusing, both in terms of cutoff (flexibility < 20% [14], 25% [12], 30% [2, 5, 19, 23], 40% [1]) and of method of assessment (standing SB [5], supine SB [5, 14, 19, 23, 24]). Moreover, traction radiographs under general anesthesia may redefine some of the included curves as non-stiff or partially stiff [26]. In this uncertain context, building solid evidence is challenging. Secondly, the retrospective design and the small sample of this study must be acknowledged. Finally, the AVR measurement according to Nash-Moe method has several limitations, especially in post-operative radiographs, in which the presence of pedicle screws can make the measurement difficult and sometimes inconsistent. In fact, while TK measurements showed good intra- and interobserver agreement (probably partially due to software aid in its measurement), the AVR measurement according to Nash-Moe did record only moderate agreement in the last follow-up X-rays. Moreover, if one considers the wide 95% CI, the intra- and interobserver agreement further decreases. The possible strengths of this study, especially considering the rarity of these cases, are the homogeneity of curves in terms of etiology (all adolescent idiopathic), topography (all main thoracic) and characteristics (number of vertebral levels involved). The present study may represent an attempt to discriminate between different severe AIS curves with the focus of developing a treatment algorithm able to highlight the indication for each corrective technique available, basing on the pathological anatomy of the deformity.

## Conclusions

The Hi-PoAD technique proved to be a valid alternative for the treatment of rigid AIS curves exceeding 90° of magnitude, with less than 25% reduction on side bending, and involving more than 5 vertebral bodies.

## Data Availability

The data that support the findings of this study are available from the corresponding author upon request.
